# Dataset of United States Incident Management Situation Reports from 2007 to 2021

**DOI:** 10.1038/s41597-023-02876-8

**Published:** 2024-01-03

**Authors:** Dung Nguyen, Erin J. Belval, Yu Wei, Karen C. Short, David E. Calkin

**Affiliations:** 1 Department of Forest and Rangeland Stewardship, Colorado State University, Fort Collins, CO 80523 USA; 2 USDA Forest Service, Rocky Mountain Research Station, 240 West Prospect, Fort Collins, CO 80526 USA; 3 USDA Forest Service, Rocky Mountain Research Station, 5775 US Highway 10 West, Missoula, MT 59808 USA; 4 USDA Forest Service, Rocky Mountain Research Station, 800 East Beckwith, Missoula, MT 59801 USA

**Keywords:** Natural hazards, Environmental sciences

## Abstract

This paper presents a unique 15-year dataset of Incident Management Situation Reports (IMSR), which document daily wildland fire situations across ten geographical regions in the United States. The IMSR dataset includes summaries for each reported day on national and regional wildfire activities, wildfire-specific activities, and committed fire suppression resources (i.e., personnel and equipment). This dataset is distinct from other wildfire data sources as it provides daily information on national fire suppression resource utilization, national and regional preparedness levels, and management priority for each region and fire. We developed an open-source Java program, IMSR-Tool, to process 3,124 IMSR reports available from 2007 to 2021 to generate this structured IMSR dataset, which can be updated when future reports become available. The dataset presented here and its future extension enable researchers and practitioners to study historical wildfire activity and resource use across regions and time, examine fire management perceptions, evaluate strategies for fire prioritization and fire resource allocation, and exploit other broader usage to improve wildfire management and response in the United States.

## Background & Summary

Wildland fire activity in the United States (US) has escalated during the last several decades, especially in the western US^1–5^. Between 1991 and 2020, US wildfire area burned (WFAB) has increased by approximately 77,700 hectares (ha) per year^6^, with the average annual WFAB since 2000 (2.8 million ha) being more than double the annual average of the previous decade (1.3 million ha during the 1990s)^7^. Larger wildfires have attracted growing attention in the US due to their harmful impacts on the economy, environment, and human health and safety^6^. Management response demands are expected to increase because of the escalating wildfire danger^8,9^, especially as more severe and larger wildfires are predicted to continue in the US until at least the late 21^st^ century^10^. Additionally, wildfire management appropriations have doubled from $3.1 billion in 2001 to $6.1 billion in 2020 in response to growing fire risk^11^.

**Fig. 1:**
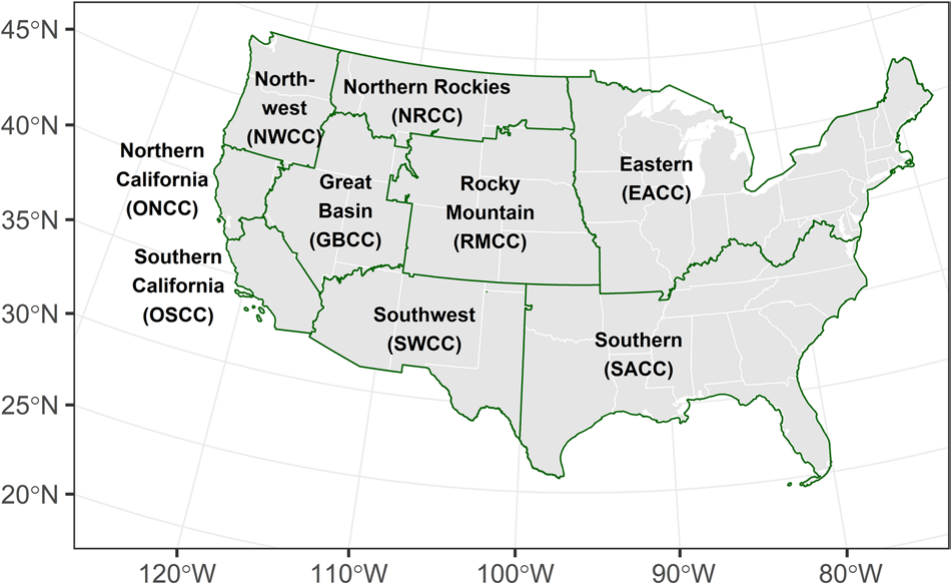
The spatial boundaries, names, and abbreviations of nine Geographic Area Coordination Centers responsible for wildfire management in the conterminous United States. Alaska (AICC; the tenth geographic area), Hawaii (part of ONCC), and Puerto Rico (part of SACC) are not included in this map.

Wildland fire management is a collaborative effort between federal, state, and local authorities. The National Interagency Fire Center (NIFC) provides the framework for interagency coordination of wildfire response among different agencies and organizations within the US wildfire response system. NIFC hosts the National Interagency Coordination Center (NICC) which provides logistical support for the national mobilization of resources (i.e., personnel and equipment) tasked with wildfire response across the country. The US has ten Geographic Area Coordination Centers (GACCs, see Fig. 1). GACCs are set up similarly to NICC, but facilitate coordination of wildfire response within their own spatial domains. NICC coordinates with the GACCs across the US to support areas of the country experiencing elevated needs for wildfire response^12,13^. While wildfire response can differ depending upon the managing agency^14^, the interagency coordination system allows all agencies involved in wildfire response to share resources with each other to best meet their land management and community protection missions. As a part of the interagency effort to provide responsive, effective, and reliable wildfire support, the National Predictive Services Program was implemented to provide decision support services to the wildland fire community at both the GACC and the NICC levels^15^. Additionally, the US Wildland Fire Applications Information Portal (WFAIP, https://www.wildfire.gov; and its predecessor FAM-IT, https://famit.nwcg.gov) was established to host a collection of applications, tools, and data services relevant to fire management.

Wildfire data play an important role in providing historical fire context, forecasting fire activity and response, and thereby improving the effectiveness and efficiency of fire management and planning^16^. For instance, wildfire data can be used to adjust suppression response strategies based on historical fire trends and optimize the allocation of firefighting resources to ongoing fires, both of which can enhance the effectiveness and efficiency of wildfire management efforts. Datasets accessible via WFAIP include those that can be used to assess historical incident management activities and firefighting resource use. These data are generally derived from the national Situation Reporting (SIT-209) and Interagency Resource Ordering Capability (IROC; previously the Resource Ordering and Status System, i.e., ROSS) applications. Data from SIT-209 and ROSS/IROC, dating back to 1999 and 2008, respectively, contain detailed information regarding daily wildfire characteristics, suppression resource requests and assignments, and other information associated with the life cycle of individual incidents. While ROSS and IROC require approval for access, the SIT-209 archive is publicly available on WFAIP. Researchers and managers have used these datasets to gain insights into drivers of wildfire activity and associated socioeconomic impacts^17,18^, suppression resource supply and demand^13,19^, and firefighting effectiveness^20,21^. However, the ROSS/IROC and SIT-209 data were not originally provided with research applications in mind. These raw data require careful preparation (i.e., cleaning, standardizing, and compiling) to be suitable for scientific research and analyses. We are unaware of any effort or intention to publish a research-ready version of the ROSS/IROC data, as they have restricted access. However, there are research-ready versions of the Incident Status Summary (ICS-209) portion of the SIT-209 data archive^22,23^ spanning 1999–2020, including linkages to agency fire reports^24^. Here, we describe a complimentary effort to generate a processed and quality-checked version of the Incident Management Situation Report (IMSR) component of the SIT-209 application.

The National Incident Management Situation Reports originated in 2000, following the establishment of the US Predictive Services. IMSRs are produced by the NICC with the goal of providing a complete and concise synopsis of on-going wildfire activity to the wildfire response community. The NICC staff produces and releases IMSRs daily during the fire season (roughly April through October) and weekly otherwise. IMSRs convey information about the risk and impact of new and ongoing wildfires in every GACC of the US, and the availability of personnel and equipment responding to those fires. Each IMSR is structured to begin with a national-level summary of wildfire activity for the reporting period (i.e., day or week), followed by a synopsis of significant wildfire activity in each GACC region. The IMSR data are used by decision makers for a variety of purposes, such as determining where to allocate scarce resources during periods of elevated fire activity. The NICC staff maintains a publicly available archive of the historical IMSRs as portable document format (PDF) files, which can be downloaded from https://famprod.nwcg.gov/batchout/IMSRS_from_1990_to_2022. This is the original source of the IMSR data that we collected and processed.

The IMSR contains three types of key information that make it a unique and valuable resource. First, it is the only publicly available dataset that contains the national and regional preparedness levels (PL), which are determined daily by national and regional fire managers. The PL, ranging from 1 to 5, indicates increasing levels of both fire danger and fire suppression resource commitments (https://www.nifc.gov/fire-information). Specific PLs may also trigger particular management activities, such as daily briefings and meetings of the National Multiagency Coordinating Group (NMAC) to coordinate, prioritize and oversee assignments of suppression resources when the national PL reaches 4. Second, while a wealth of detailed incident-specific information is available in the broader SIT-209 dataset, the IMSR is the only public source of the daily/weekly wildfire management prioritization. The order of GACCs reported in the IMSR indicates the priority rank given to each GACC by the NMAC. Similarly, wildfires occurring in each GACC are presented in descending priority order. Finally, the IMSR exclusively provides a daily summary of national fire suppression needs and resource utilization, including number of fires, cumulative fire size, number of personnel, crews, engines, and helicopters committed to all fires reported in each GACC. There is currently no other publicly accessible data source that provides the number of resources assigned to all fire incidents at this temporal scale. While ROSS and IROC allow trained and experienced users to create similar daily counts^13,19^, the data can be time-consuming to process, and some data on suppression response for smaller fires may be missing.

The IMSR is a valuable data source for both fire managers and researchers^25^, and we have seen efforts to obtain and use several pieces of IMSR information in research including the PL^26,27^ and suppression resource use^28–30^. However, we are unaware of other efforts to generate a research-ready version of the IMSR archive, which would support broader use of these data. The greatest challenge of extracting the IMSR data as structured content comes from the file format, as archived IMSR data are only available in PDF reports. Given the sheer volume of IMSR PDFs archived over the past three decades (1990–2022), extracting information manually from these files is a tedious, time-consuming, and error-prone task. This has motivated our effort to develop a process to automatically extract information from the raw IMSR data. Our goal is to produce a structured IMSR dataset from the historical archive, which can serve as a vital resource for wildfire researchers and managers in studying historical wildfire activity, suppression resource use and prioritization. It has the potential to offer valuable evidence and insights to improve future wildfire management and planning.

In this paper, we present the structured dataset mined from historical IMSRs, which covers a 15-year period from 2007 to 2021. We chose this period because the content and format of IMSR underwent significant changes in 2007, and since then, they have remained relatively consistent. Our focus is to provide a version of IMSR data as shown in the original reports, while also addressing issues such as typos or non-standardized terms. We developed an open-source Java program to automatically extract information from historical IMSR PDF reports. This program is also capable of extracting future IMSR PDFs to extend the dataset beyond the time range in this paper, provided that the report format does not change substantially. We further demonstrated the usefulness of our dataset by linking it back to the SIT-209 data, which have been of increasing use in wildfire research and management applications. By presenting this well-structured IMSR dataset, we aim to benefit not only researchers and managers but also the general public who are interested in accessing and utilizing IMSR information.

## Methods

### Raw data collection

According to the 2021 document entitled “Understanding the IMSR” from Predictive Services (no longer accessible online but included in our data repository^31^), IMSR reports are generated daily during the fire season at the National PL 2 and above, and weekly (often on Fridays) at the National PL 1. It is important to note that, according to the latest 2023 National Interagency Mobilization Guide^12^, IMSRs are issued daily when the National PL reaches 3 or higher. An IMSR report may also be produced on any day when there is significant wildfire activity or resource mobilization^12^. Wildfires classified as significant must burn at least 40 ha (100 acres) in timber or slash fuel types, 121 ha (300 acres) in grass or brush fuels, or are otherwise managed by a Type 1 or Type 2 Incident Management Team^12^. Once a fire is included in an IMSR, it will continue to be reported in future IMSRs until it is contained, personnel assigned drops below 100, or the fire typically diminishes^12^.

Historical IMSRs were archived as PDF files at the National Wildfire Coordinating Group website at https://famprod.nwcg.gov/batchout/IMSRS_from_1990_to_2022. We have downloaded all 3,124 PDFs from the website for the 15-year period from 2007 to 2021 (Table 1). These served as raw data for further processing and extraction.

**Table 1:** Number of IMSR reports available for the period 2007–2021.

Year/Month	Jan	Feb	Mar	Apr	May	Jun	Jul	Aug	Sep	Oct	Nov	Dec
2007	3	3	7	21	31	30	31	31	29	17	12	4
2008	4	8	4	14	31	30	31	31	30	31	10	4
2009	5	4	18	22	31	30	31	31	30	26	11	4
2010	4	3	4	15	29	30	31	31	30	30	26	5
2011	4	11	31	30	31	30	31	31	30	28	13	4
2012	4	4	3	0	31	30	31	31	30	21	16	12
2013	4	4	5	4	15	30	31	31	30	3	5	3
2014	4	4	4	4	19	30	31	31	30	7	3	4
2015	3	4	4	4	5	30	31	31	30	6	4	5
2016	2	4	4	5	4	30	31	31	30	4	23	10
2017	4	3	5	3	17	30	31	31	30	31	4	19
2018	4	4	5	19	31	30	31	31	30	15	12	3
2019	0	4	5	4	5	26	31	31	29	25	9	4
2020	3	3	4	8	21	30	31	31	30	31	13	5
2021	4	3	4	21	21	30	31	31	30	21	4	5

### Procedure to process the raw data

**Fig. 2:**
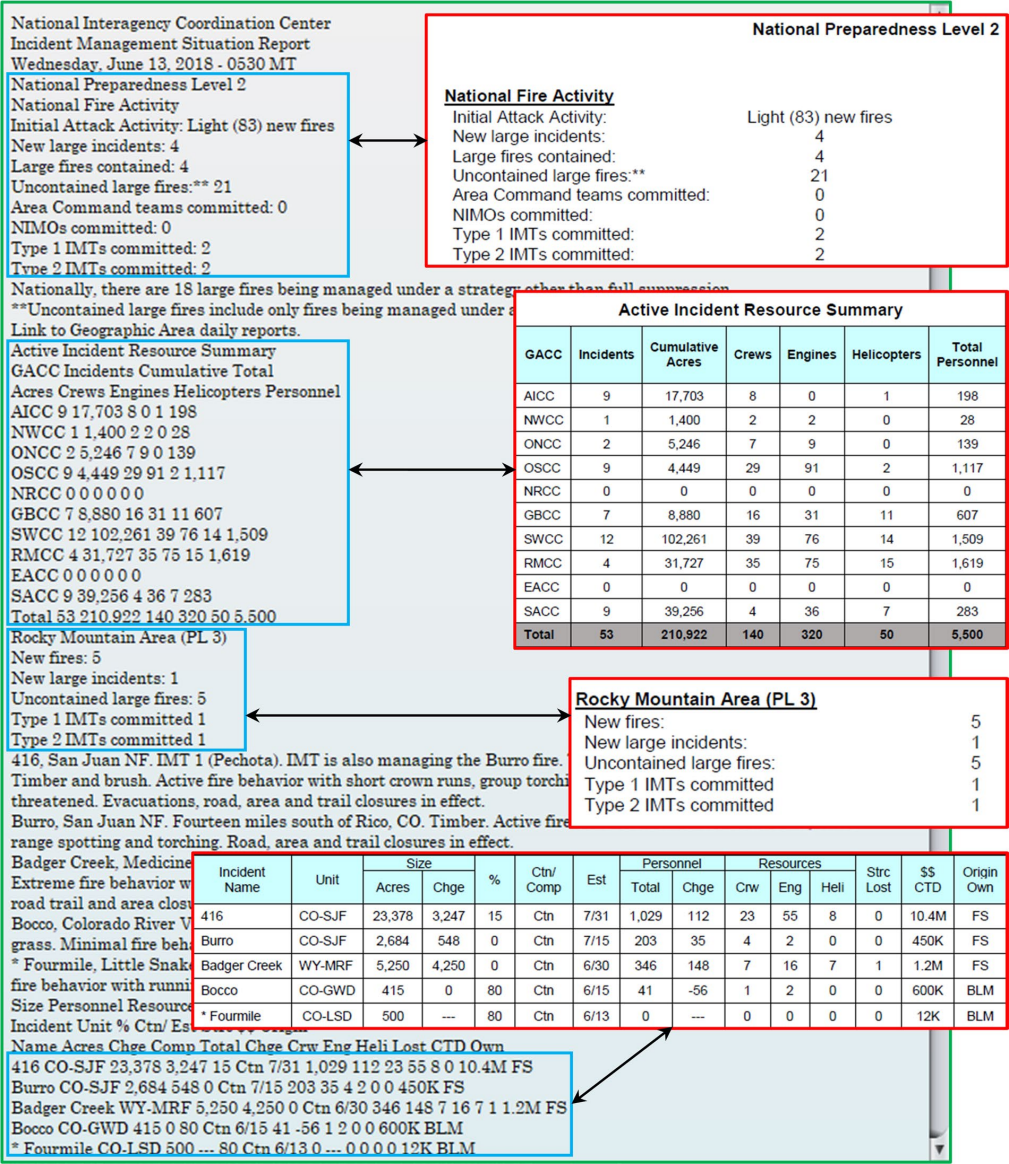
Example of PDF to TXT conversion by IMSR-Tool (green-border box). The four red-border boxes are screenshots taken from an example IMSR PDF to illustrate the four data categories to be pulled out. Their corresponding texts converted into the TXT file are highlighted in the four text blocks with blue borders, which will be used for further data processing and extraction based on keywords and text patterns recognition.
- Step 2 – text file processing: Text contents in each TXT file were filtered and split into text blocks based on keywords and text patterns. Keywords are phrases that remain unchanged across different IMSR reports, such as those presented in the boxes with blue or red borders in Fig. 2. Text patterns can be identified from table data included in the IMFR reports. For example, data in each row of a specific table in an IMSR report often have a fixed number of words presented in the same line of the corresponding TXT file, and some text strings at certain positions of each line in an IMSR table contain only numeric characters (e.g., the two tables shown in Fig. 2).
- Step 3 - data extraction and cleaning: Data associated with keywords and text patterns (i.e., texts found next to certain keywords or with certain recognized text patterns) were extracted and organized into different data categories (Fig. 3). Subsequently, the extracted data were cleaned and formatted (Table 2).

A Java program (IMSR-Tool^32^) was developed with a graphical user interface (GUI) to support the process of creating a structured dataset from raw IMSR data. The process includes four steps:

- Step 1 - file conversion: Raw PDF reports were converted into text files using XPDF (https://www.xpdfreader.com), an open-source Java library integrated in the IMSR-Tool. This Java library can recognize and extract texts from PDFs and save the information to text (TXT) files with a consistent format (Fig. 2).

**Fig. 3:**
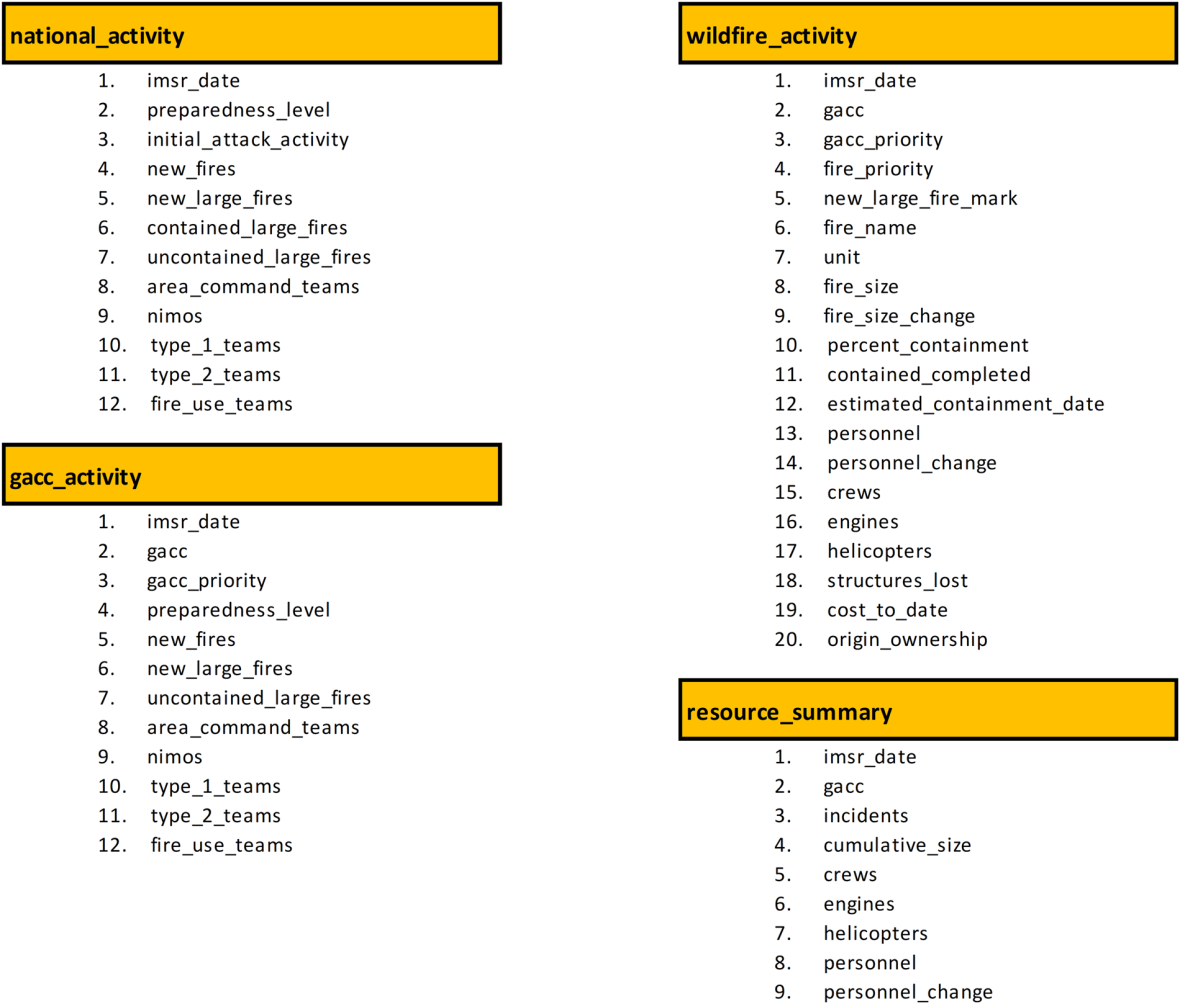
Four data categories (tables) and their corresponding data elements (fields) extracted by IMSR-Tool. Field names were obtained from the raw IMSR PDFs and slightly modified to be concise and self-explained.

**Table 2:** Data cleaning and formatting implemented by IMSR-Tool.

Cleaning and formatting action (number of instances)
**For all tables of the IMSR dataset**
- Reformatting all texts into UNICODE standard encode. - Capitalizing all texts - Standardizing terms: replacing N/A by NA (7579), and replacing N/R by NR (3) - Replacing multiple consecutive spaces with a single space - Removing all leading and ending spaces - Removing all spaces surrounding slash or hyphen - Removing commas in numbers, such as 1,000 to 1000 - Removing duplicated records (89, including 86 duplications on 2020-07-19 and one duplication on each of the following dates: 2021-09-15, 2016-11-09, 2007-03-02)
**For the wildfire_activity table of the IMSR dataset**
- Moving the asterisk in “fire_name” to another data field “new_large_fire_mark” - Typos including–(156), ---- (51), - (3),… (3), __ (1), ___ (1) were replaced by– - Typos including grave accent (14) and equal sign (2) were removed - Typos in “estimated_containment_date” including ÚNK (13) and UKN (4) were replaced by UNK - Typos in “contained_completed” including CNT (4) and CTN. (1) were replaced by CTN - Typo in “cost_to_date” including NF (4) and NRK (1) were replaced by NR - Removing the dollar sign in “cost_to_date” (3) - Revising “cost_to_date” values to adhere to the format of a number followed by a character K or M, which respectively represent thousand or million dollars (141). By comparing the “cost_to_date” values of the same fire reported in multiple IMSRs, 95 records were corrected by adding K or M. The remaining 46 records were left unchanged because each of those fires was reported by only a single IMSR. - Correcting non-date values in “estimated_containment_date” (13) - Correcting non-integer values in “fire_size” (30), “fire_size_change” (2), “percent_containment” (2), “personnel” (4), “personnel_change” (1), and “structures_lost” (11) - Correcting numbers representing “origin_ownership” (2)

**Fig. 4:**
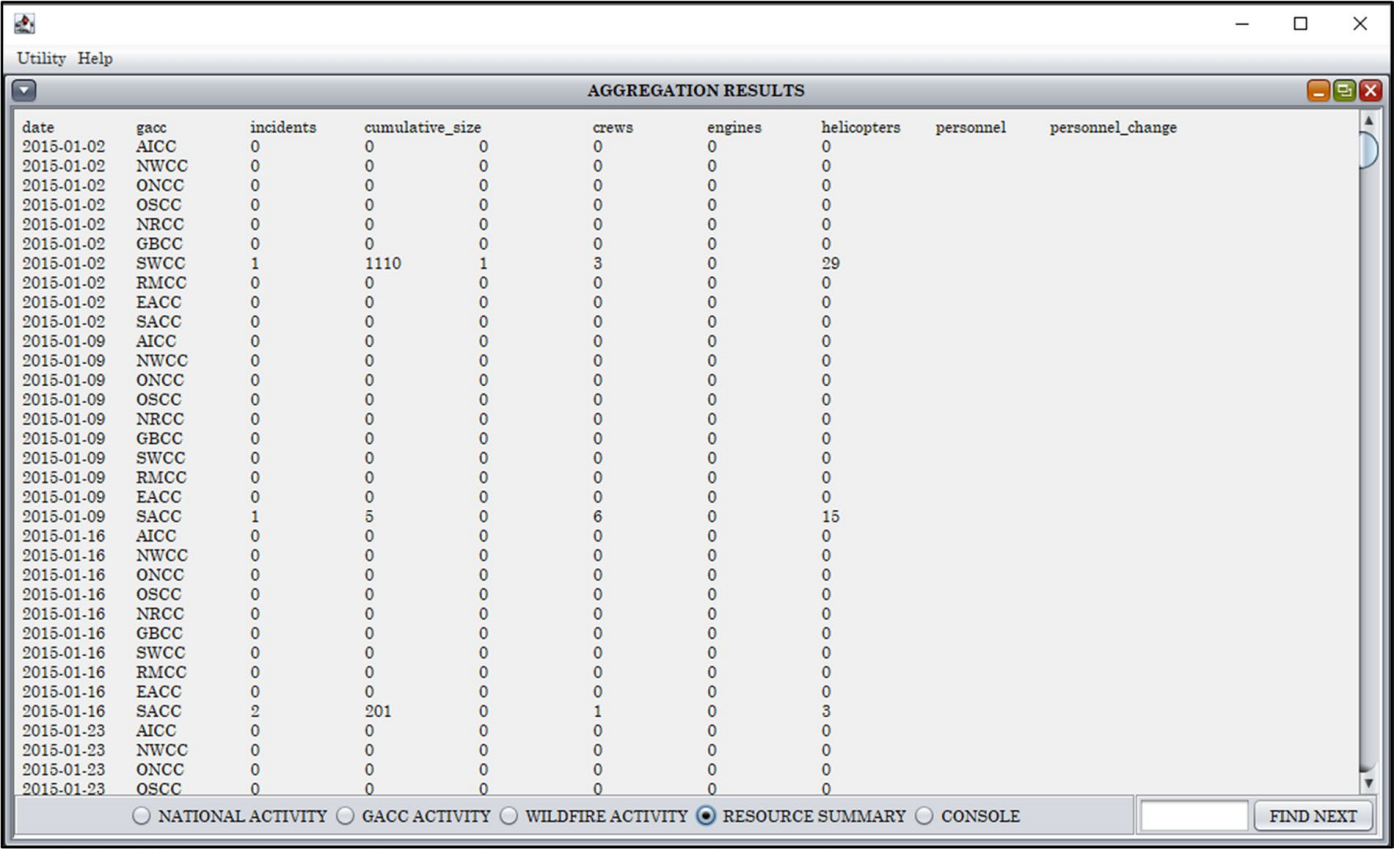
A screenshot of the IMSR-Tool’s graphical user interface that enables user to explore and export data extraction results.

Note that the underscored numbers represent typos (- and NRK) that were manually corrected, not by IMST-Tool. Each special character or abbreviation has a specific meaning, such as NR for “not reported”, NA for “not available”, UNK for “unknown”; * representing “a new large fire”;–indicating “the lack of information for a new fire”. More details can be found in the “Understanding the IMSR” document (no longer accessible online, but included in our data repository^31^).
- Step 4 – data export: Finally, data can be examined and exported through the built-in GUI functions of the IMSR-Tool (Fig. 4). Structured data are presented as tables with tab delimited texts in the GUI. Data examination, such as searching by keywords or navigating view between daily records, is also supported by the GUI. Exporting the data to tab delimited text files can be done through standard copy of the GUI’s tables and paste to external applications.

## Data Records

The dataset produced from this study contains IMSR information in the US from 2007 to 2021. It consists of three tables that separately store daily wildfire activity at three different levels (national, regional, specific wildfire) and a summary table of active fire suppression resource use for all fires in each GACC during each day (Table 3). IMSR tables cover data fields within specific categories (Tables 4–7) and may not reflect the complete set of information related to each fire. For example, details about suppression resources such as airtankers are not reported by IMSR. However, such detailed information can be found in SIT-209 through cross-referencing (joining) IMSR and SIT-209, which will be demonstrated in the next section of this paper. Information in some tables may not be available for certain time periods if they were not reported by IMSR (Table 8). All dataset tables are stored in comma-delimited files (national_activity.csv, gacc_activity.csv, wildfire_activity.csv, resource_summary.csv) and within separate sheets of a single Excel file (2007–2021-IMSR-1.06.xlsx) for convenient usage. We deposited the dataset at figshare^31^.

**Table 3:** Tables included in the IMSR dataset. More details of the data fields are presented in Tables 4–7.

Table name	Table description	Fields	Records
national_activity	Synopsis of national wildfire activity that occurred since the last IMSR report was produced	12	3,124
gacc_activity	Synopsis of wildfire activity that occurred in a GACC since the last IMSR report was produced	12	16,516
wildfire_activity	Statistical information of every large fire burning in a GACC	20	88,211
resource_summary	Summary of active fires and acres burning in each GACC and the resources committed to these incidents	9	13,530

**Table 4:** Data fields in the “national_activity” table.

Data field name	Data field description	Data type
imsr_date	IMSR report date	Date (YYYY-MM-DD)
preparedness_level	National preparedness level (https://www.nifc.gov/fire-information)	Integer (1 to 5)
initial_attack_activity	National initial attack activity level	Text (Light, Moderate, Heavy)
new_fires	New fires reported nationwide	Integer
new_large_fires	New significant fires reported nationwide	Integer
contained_large_fires	Contained significant fires reported nationwide	Integer
uncontained_large_fires	Uncontained significant fires reported nationwide	Integer
area_command_teams	Area command teams assigned nationwide	Integer
nimos	National incident management organizations assigned nationwide	Integer
type_1_teams	Type 1 incident management teams assigned nationwide	Integer
type_2_teams	Type 2 incident management teams assigned nationwide	Integer
fire_use_teams	Fire use teams assigned nationwide	Integer

**Table 5:** Data fields in the “gacc_activity” table.

Data field name	Data field description	Data type
imsr_date	IMSR report date	Date (YYYY-MM-DD)
gacc	Abbreviated name of a Geographic Area Coordination Center	Text (4 characters)
gacc_priority	Priority ranking of the GACC at the national level	Integer
preparedness_level	GACC preparedness level (https://www.nifc.gov/fire-information)	Integer (1 to 5)
new_fires	New fires in the GACC	Integer
new_large_fires	New significant fires in the GACC	Integer
uncontained_large_fires	Uncontained significant fires in the GACC	Integer
area_command_teams	Area command teams assigned in the GACC	Integer
nimos	National incident management organizations assigned in the GACC	Integer
type_1_teams	Type 1 incident management teams assigned in the GACC	Integer
type_2_teams	Type 2 incident management teams assigned in the GACC	Integer
fire_use_teams	Fire use teams assigned in the GACC	Integer

**Table 6:** Data fields in the “wildfire_activity” table.

Data field name	Data field description	Data type
imsr_date	IMSR report date	Date (YYYY-MM-DD)
gacc	Abbreviated name of a Geographic Area Coordination Center	Text (4 characters)
gacc_priority	Priority ranking of the GACC at the national level	Integer
fire_priority	Priority ranking of the fire at the GACC level	Integer
new_large_fire_mark	Asterisk (*) indicates a new large fire	Character (*)
fire_name	Fire name	Text
unit	Abbreviated name of the agency responsible for managing the fire	Text
fire_size	Fire size in acres	Integer
fire_size_change	Change in fire size in acres since last report	Integer
percent_containment	Proportion of the fire that has been contained	Integer (0 – 100)
contained_completed	Progress towards completion of the incident objectives	Integer (0 – 100)
estimated_containment_date	Estimated date for fire containment or completion	Date (either MM/DD, MM/D, M/DD, M/D)
personnel	Number of personnel assigned to the fire	Integer
personnel_change	Change in number of personnel assigned to the fire since last report	Integer
crews	Number of crews assigned to the fire	Integer
engines	Number of engines assigned to the fire	Integer
helicopters	Number of helicopters assigned to the fire	Integer
structures_lost	Number of structures destroyed by the fire	Integer
cost_to_date	Estimated suppression cost to date. The ending character K or M represents thousand or million USD, respectively.	Double (ending with K or M)
origin_ownership	Origin ownership whose land the fire started on	Text

**Table 7:** Data fields in the “resource_summary” table.

Data field name	Data field description	Data type
imsr_date	IMSR report date	Date (YYYY-MM-DD)
gacc	Abbreviated name of a Geographic Area Coordination Center	Text (4 characters)
incidents	Number of active incidents in a GACC reported by the SIT-209 application, regardless of incident type or size	Integer
cumulative_size	Acres burned on all active incidents reported by the SIT-209 application. Active incidents may or may not meet large (or significant) fire criteria.	Double
crews	Number of crews assigned to all active incidents in a GACC, as reported by the SIT-209 application	Integer
engines	Number of engines assigned to all active incidents in a GACC, as reported by the SIT-209 application	Integer
helicopters	Number of helicopters assigned to all active incidents in a GACC, as reported by the SIT-209 application	Integer
personnel	Number of fire personnel assigned to all active incidents in a GACC, as reported by the SIT-209 application	Integer
personnel_change	Change in number of fire personnel in a GACC, compared to the previously published IMSR	Integer

**Table 8:** Dataset notes.

Note and description
**Different reported regions**
- Prior to 2015, IMSRs reported information for two sub-areas of the Great Basin (GBCC) including Eastern Great Basin (EBCC) and Western Great Basin (WBCC). Since 2015, IMSRs only reported information for the entire GBCC as a whole.
**Unreported data**
- “resource_summary” table was not reported in years prior to 2015 - “fire_use_teams” in “national_activity” was not reported on dates after 2009-03-20 - “fire_size_change” in “wildfire_activity” was not reported on dates prior to 2007-05-28 - “personnel_change” in “wildfire_activity” was not reported on dates prior to 2007-05-28 - “contained_completed” in “wildfire_activity” was not reported on dates prior to 2015-01-02 - “personnel_change” in “resource_summary” was not reported on dates prior to 2021-04-06

## Technical Validation

We employed systematic sampling^33^ to assess the accuracy of each of the four IMSR tables. This sampling method combines randomness with a degree of control for selecting samples. Systematic sampling is suitable for validating our data because: (1) the population size of each table is known (see the second column of Tables 9), and (2) the varying IMSR report dates and the diverse numbers of GACC regions and fires reported daily (or weekly) can prevent systematic sample-selection from encountering a specific data pattern that may exist.

**Table 9:** Accuracy assessment for the IMSR tables.

IMSR table name	All records within 2007–2021 period (Population size)	Total number of sampled records (Sample size)	Accurately extracted records	Inaccurately extracted records	Accuracy (%)
national_activity	3,124	343	343	0	100
gacc_activity	16,516	376	376	0	100
wildfire_activity	88,211	383	383	0	100
resource_summary	13,530	374	374	0	100

Note that “Total number of sampled records” is the sum of “Accurately extracted records” and “Inaccurately extracted records”. “Accurately extracted records” represents the number of sampled records that have all data fields matched entirely when comparing results between automatic extraction (by IMSR-Tool) and manual extraction. “Accuracy” is calculated by 100 times the “Accurately extracted records” divided by the “Total number of sampled records”.

To employ systematic sampling, we first used an online tool (https://www.asqa.gov.au/resources/tools/validation-sample-size-calculator) to calculate the required sample size for each table (as listed in the third column of Table 9) based on its population size, a 95% confidence level, and a 5% margin of error level. A selection interval “k” was calculated for each table by dividing the population size by the sample size. Samples, each is represented by a row in the data table, would then be selected from the population at positions determined by a random start between 1 and k and every k^th^ increment thereafter. Using a fixed interval for selecting samples can ensure that the population will be evenly sampled. This is necessary to mitigate clustered selections and adequately capture the changes in reporting content and format of IMSR over time, thereby preventing potential biases during the accuracy validation.

For the selected samples associated with each IMSR table, we compared results between automatic data extraction (by IMSR-Tool) and manual data extraction to calculate the accuracy of the automatic extraction method. The detailed sampling and validation process were included in our data repository^31^ (refer to the file “Technical-Validation-IMSR-1.06.xlsx”), and were summarized here in Table 9. The comparison results show high quality of data extraction using IMSR-Tool, with 100% accuracy observed for every table.

Note that when comparing results between automatic extraction and manual extraction, we disregarded differences due to data cleaning and formatting implemented by IMSR-Tool (as listed in Table 2). We encountered such differences while validating two IMSR tables:

- wildfire activity: There are 15 records (rows) where data values are different due to abbreviation format (i.e., N/A was replaced by NA), 1 record (2019-08-03, Devil’s Elbow) with a difference in the apostrophe format (IMSR-Tool converted the original apostrophe format into UNICODE), and 1 record where a typo was programmatically fixed (i.e., 7.555 was replaced by 7555 as the size of the 2011-03-11 Emin fire). All those 17 records were considered to be accurate.
- resource_summary: There are 9 records where GACC names are different due to programmatic format (i.e., GACC names used before 2016 were replaced by their corresponding new names used since 2016, such as replacing AKCC by AICC). All those 9 records were considered as accurately extracted by IMSR-Tool.

## Usage Notes

As previously mentioned, the dataset covers a 15-year period from 2007 to 2021. However, the IMSR-Tool presented in this paper is able to process new data in the future to add results of subsequent years to the existing dataset. We have established a long-term support plan for updating the tool to mine future IMSR reports in case the PDF file format may change. Note that mining IMSRs prior to 2007 is not supported due to inconsistencies in both reporting content and format.

### Potential usage

**Fig. 5:**
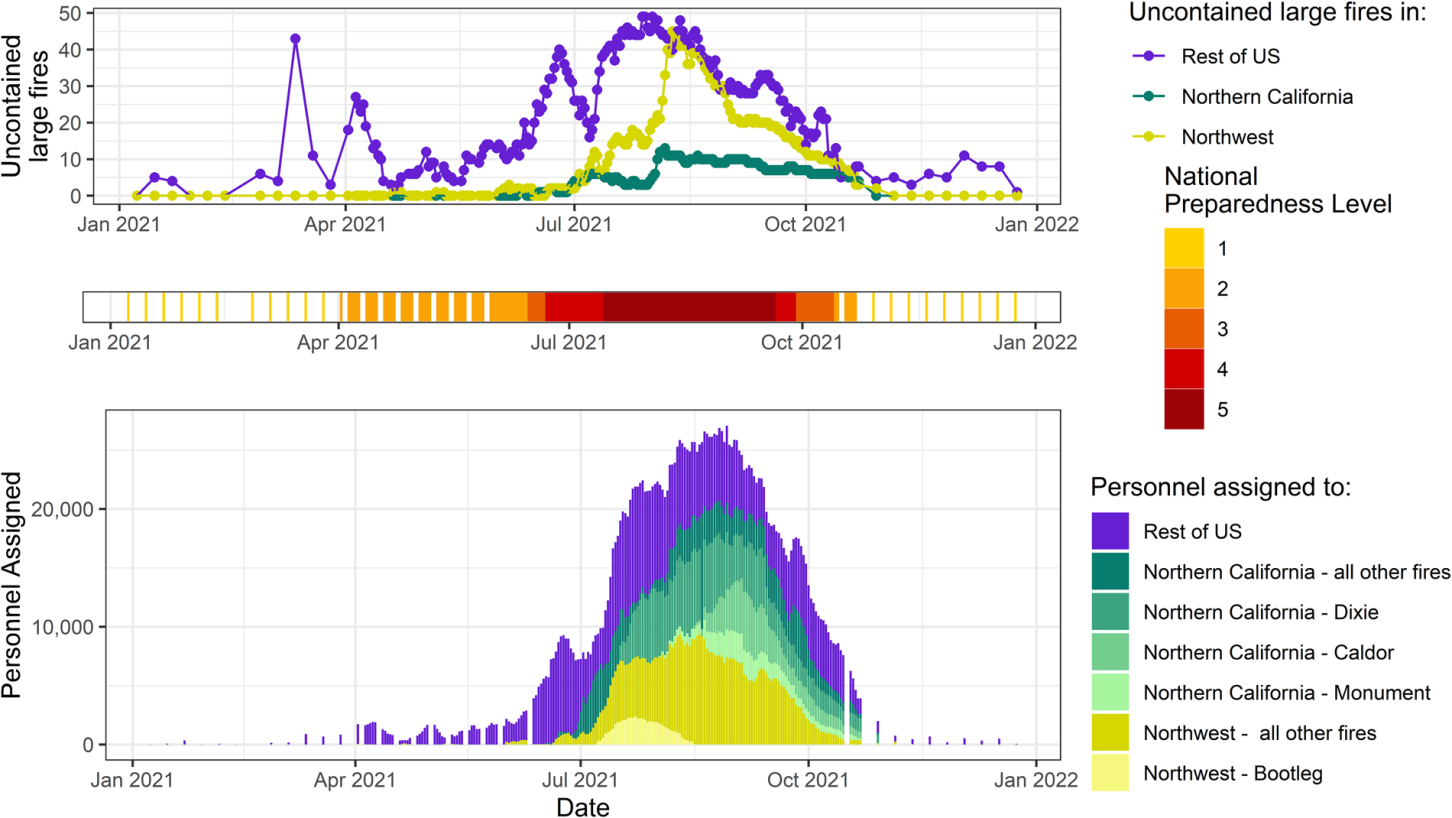
Daily national preparedness level, number of large fires, and number of personnel assigned, looking specifically at two US regions (Northern California and Northwest) and the four largest-size fires (Dixie, Bootleg, Monument, and Caldor) occurring within these regions in 2021.

This dataset provides a unique combination of both wildfire activity and suppression resource assignments, which can be used to provide historical wildfire activity context across regions of the United States. Statistics and visual examinations based on IMSR data (such as Fig. 5) can provide insights for wildfire management and trigger compelling questions for fire research. The IMSR holds valuable fire data that can serve as inputs for building a variety of quantitative fire models to inform wildfire management decision making. For example, past resource allocation patterns within IMSR can be used in a resource request forecasting model to predict future resource needs, which facilitate proactive fire planning. Historical cost trends derived from IMSR can help develop a cost estimation model to predict firefighting expenses for each future wildfire event, enabling fire agencies to improve budget planning and allocation. The IMSR data holds significant potential for a broader range of applications beyond the mentioned examples. It can serve as a foundational element in these applications, driving decisions to improve wildfire management outcomes.

### Data connection

The dataset produced here has the potential to connect to several major wildland fire data sources of United States such as ROSS, IROC, and SIT-209. While ROSS and IROC remain inaccessible to the general public, the SIT-209 data archive is publicly available via WFAIP (https://www.wildfire.gov/application/sit209). Here, we demonstrated how the IMSR data could be connected to the SIT-209 data.

**Fig. 6:**
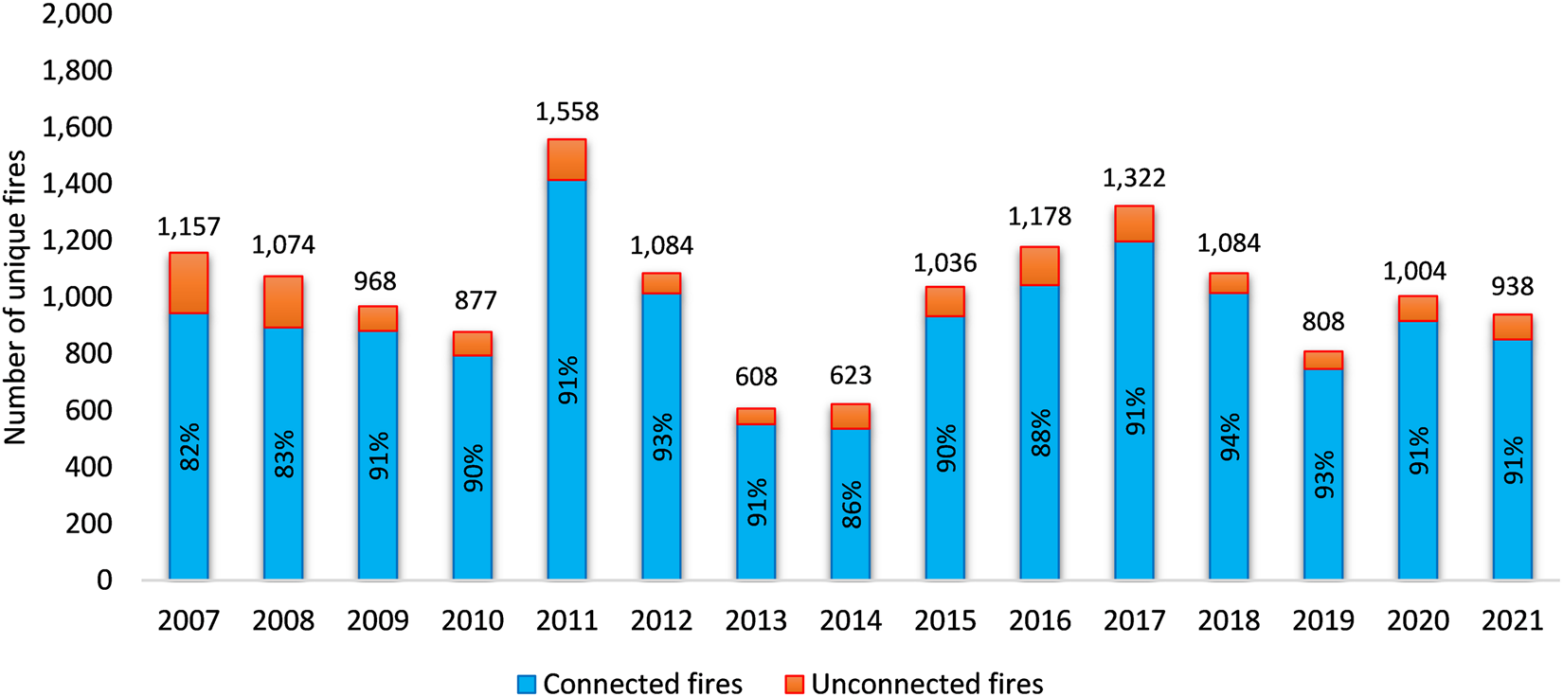
Connecting unique fires in IMSR and in SIT-209 based on matching unformatted fire names. The total number of unique IMSR fires for each year is shown above each column.

**Fig. 7:**
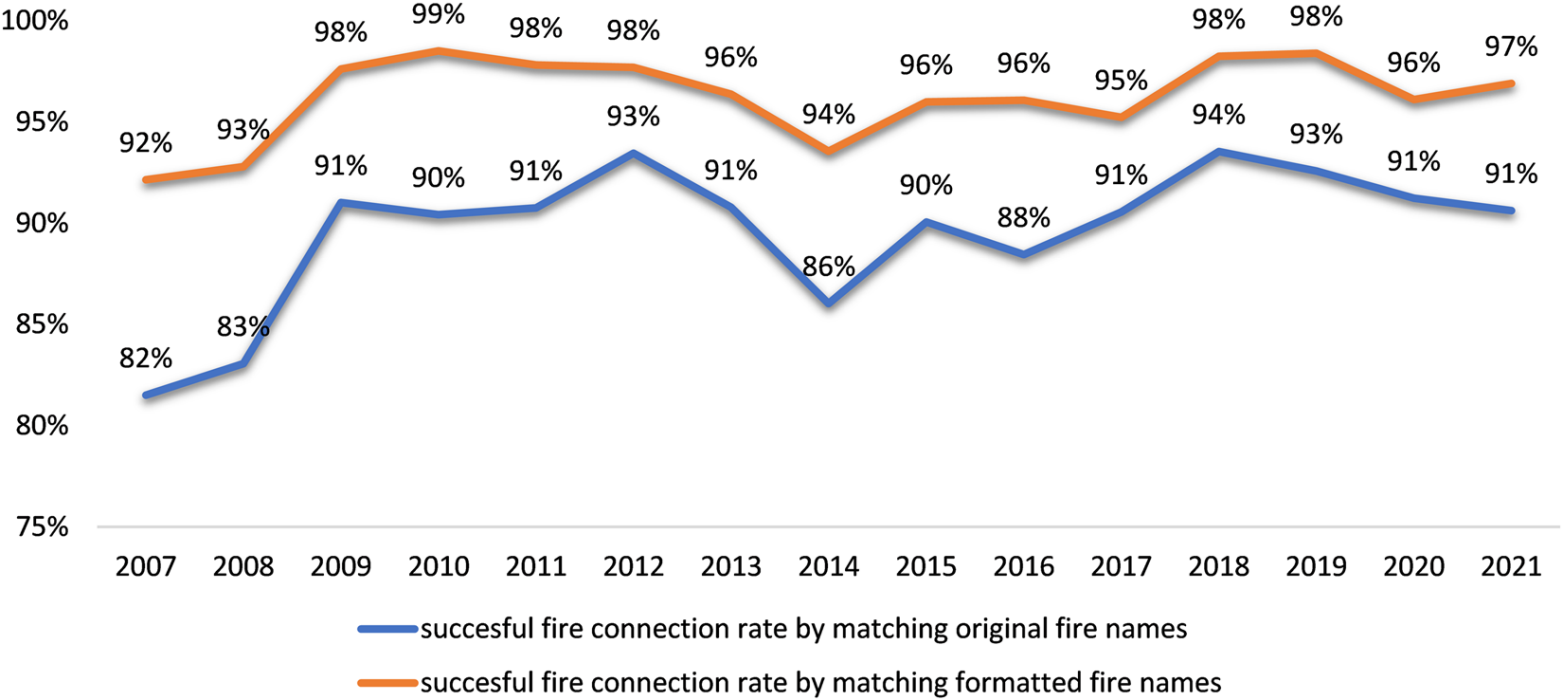
Comparison of results from connecting unique fires in IMSR and in SIT-209 by matching unformatted fire names or by matching formatted fire names.

Figure 6 shows the result of connecting unique fires in IMSR to SIT-209 where fire names are required to be exactly matched. To understand the potential reasons of mismatching when joining between IMSR and SIT-209, we randomly picked the year 2018 for scrutinization. There were 6% IMSR records including 70 incidents that could not find a match in SIT-209. Among those 70 IMSR incidents, two did not exist in SIT-209, while the other 68 incidents could be found in SIT-209 with unmatched names. Common reasons for mismatching include missing the word “FIRE” in the incident name (39/68) such as DUNCAN vs DUNCAN FIRE, spacing issue (9/68) such as ROSE BUD vs ROSEBUD, typos (7/68) such as COFFEE RIDGE vs COFFEY RIDGE, and other issues causing slightly name difference (13/68) such as ROAD vs RD, ROAD vs LANE, SPRING vs SPRINGS, etc. Formatting fire names from both IMSR and SIT-209 before joining can improve the successful connection rate and reduce the post-connection linking effort. For example, by trimming all the spaces and special characters while keeping only the alphanumeric characters and removing the word “FIRE” from incident names in both data sources, the rates of successful connection increased by 4–10% (Fig. 7).

**Fig. 8:**
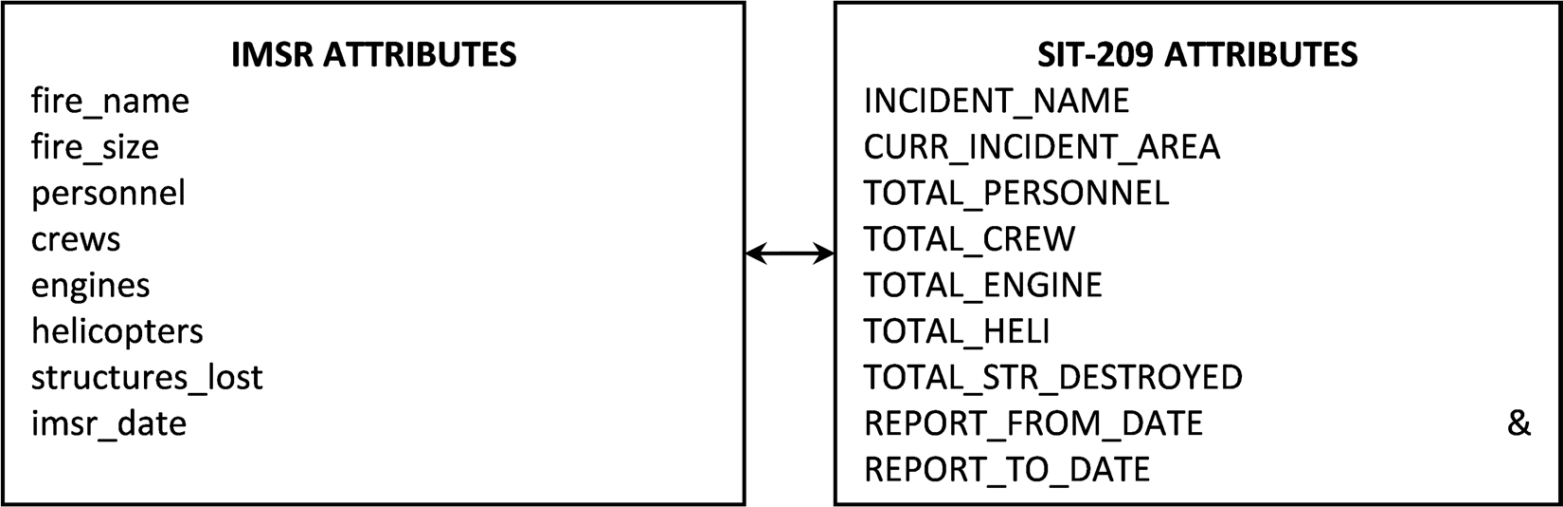
Matching daily fires between IMSR and SIT-209 based on eight different fire attributes.

**Fig. 9:**
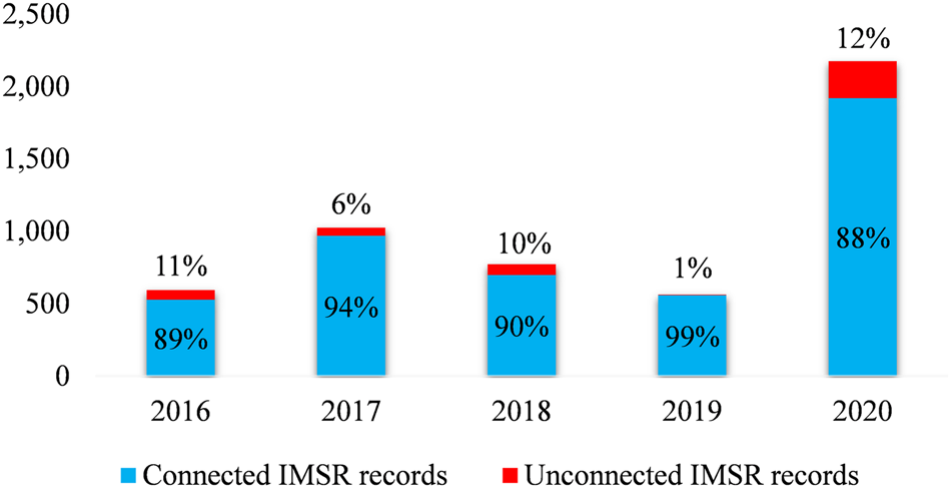
Number of connected and unconnected IMSR fire records when joining to SIT-209. Four fields (TOTAL_PERSONNEL, TOTAL_CREW, TOTAL_ENGINE, TOTAL_HELI) were not used for joining the 2019 data because of unavailable information in the 2019 SIT-209 data.

Connecting IMSR and SIT-209 data by incident names can provide an overview of incidents that exist in both datasets. However, for practical usage, a more detailed connection to link daily records would be needed. A specific fire incident may have its corresponding attributes (e.g., fire size, fire resource counts) changed daily during its life cycle. And therefore, to link the daily incident records we will need to match several other fire attributes in addition to matching the fire name. Figure 8 illustrates an example of using eight different fire attributes to join the daily fire records from IMSR and SIT data. Connection results were also illustrated for five years between 2016 and 2020 (Fig. 9).

### Non-fire incidents

In addition to wildfires, non-fire incidents were also reported in IMSR when significant fire resources were committed. IMSR does not contain any information to clearly identify incident type. However, this information could be obtained from SIT-209. Table 10 shows the result of identifying incident types through joining all unique incidents in IMSR to SIT-209 based on the formatted incident names. Across 15 years, we found 89.3–98.1% of the unique incidents reported in IMSR were wildfires, while less than 2.9% annually were non-fire incidents such as hurricanes, storms, floods, tornados, prescribed fires, and complexes. Both of those percentages could be higher by taking some portions from the 1.5–7.9% of the IMSR incidents with unidentified types due to unsuccessful join between IMSR and SIT-209.

**Table 10:** Unique IMSR incidents and their types identified through joining IMSR to SIT-209 by matching formatted incident names.

Year	All incident (count)	Wildfire (count)	Other (count)	Unidentified (count)	Wildfire (%)	Other (%)	Unidentified (%)
2007	1,146	1,023	33	90	89.3	2.9	7.9
2008	1,068	973	18	77	91.1	1.7	7.2
2009	964	939	2	23	97.4	0.2	2.4
2010	872	855	4	13	98.1	0.5	1.5
2011	1,551	1,517	0	34	97.8	0.0	2.2
2012	1,082	1,056	1	25	97.6	0.1	2.3
2013	607	582	3	22	95.9	0.5	3.6
2014	620	580	0	40	93.5	0.0	6.5
2015	1,021	980	0	41	96.0	0.0	4.0
2016	1,171	1,117	8	46	95.4	0.7	3.9
2017	1,320	1,233	24	63	93.4	1.8	4.8
2018	1,078	1,037	22	19	96.2	2.0	1.8
2019	805	784	8	13	97.4	1.0	1.6
2020	999	945	15	39	94.6	1.5	3.9
2021	936	881	26	29	94.1	2.8	3.1
All years	15,204	14,502	164	574	95.2	1.1	3.8

Note: “Other” represents non-wildfire incidents, “Unidentified” represents incidents in IMSR but not found in SIT-209 through joining and therefore their types could not be identified.

## Data Availability

The dataset described in this paper is available at figshare^31^ under the Creative Commons Attribution 4.0 license (http://creativecommons.org/licenses/by/4.0). This license permits the use, sharing, adaptation, distribution and reproduction in any medium or format, as long as you give appropriate credit to the original authors and the source. The dataset was generated by IMSR-Tool, an open-source Java program that is accessible via zenodo^32^. The latest release of IMSR-Tool (version 1.06) includes a runnable desktop application and a user manual that are publicly available at https://github.com/thumit/IMSRtool/releases/tag/1.06. IMSR-Tool is licensed under the GNU General Public License version 3 or later (GNU-GPL3, http://www.gnu.org/licenses), which allows users to freely download, use, distribute, and modify the tool and its source code, given that the modified tool and source-code must be released to the public under the same GNU-GPL3 license.
